# Supplementary material for: Dynamic transcriptomic profiles of zebrafish gills in response to zinc supplementation
Source: BMC Genomics. 2010 Oct 11;11:553. doi: 10.1186/1471-2164-11-553 (PMC3091702; doi:10.1186/1471-2164-11-553)
Supplement: Additional file 2 — Interactive Direct Interaction Network representing the molecular interactions between zinc, copper, iron, calcium and proteins encoded by transcripts changed by zinc supplementation. Mini web-site containing index.html and hyperlinked pages in subdirectory describing a Direct Interaction Network automatically generated based on curated interactions contained within the proprietary PathwayArchitect database. Ovals represent proteins and the circles symbolize metal ions. Objects are coloured by their abundance in zebrafish at the time-point they were significantly different from the control is a scale from -4 fold (dark green) to +4 fold (dark red). Where significant differences were found at more than one time-point, the colour overlay shows expression at the first instance. Dark blue squares denote 'binding', and light blue squares 'expression'; green squares stand for 'regulation', green diamonds for 'metabolism', and green circles for 'promoter binding'. Arrow heads indicate directionality of the interaction where annotated. All nodes and edges can be further interrogated by selecting the relative area of the image. [file 1471-2164-11-553-S2.zip › PathwayArchitect Zn xs DIN/94948.html]

# PROTEIN: LYZ

|  |  |
| --- | --- |
| Name | LYZ |
| Type | PROTEIN |
| Description | lysozyme (renal amyloidosis) |
| Note | LYZ encodes human lysozyme, whose natural substrate is the bacterial cell wall peptidoglycan (cleaving the beta[1-4]glycosidic linkages between N-acetylmuramic acid and N-acetylglucosamine). Lysozyme is one of the anti-microbial agents found in human milk, and is also present in spleen, lung, kidney, white blood cells, plasma, aliva, and tears. Missense mutations in LYZ have been identified in heritable renal anyloidosis. |
| Alias | LZM |
|  | lysozyme |
|  | LYZ |
|  | 1,4-beta-N-acetylmuramidase C |
|  | Lysz |


---

|  |  |
| --- | --- |
| GO Component | extracellular space |
|  | mitochondrion |
|  | extracellular region |


---

|  |  |
| --- | --- |
| GO ID | GO:0003796 |
|  | GO:0005615 |
|  | GO:0019835 |
|  | GO:0016998 |
|  | GO:0003824 |
|  | GO:0042742 |
|  | GO:0016798 |
|  | GO:0016787 |
|  | GO:0005576 |
|  | GO:0005975 |
|  | GO:0005739 |


---

|  |  |
| --- | --- |
| MIM | MIM:105200 |
|  | MIM:153450 |


---

|  |  |
| --- | --- |
| Connectivity | 16 |


---

|  |  |
| --- | --- |
| Entrez ID | 25211 |
|  | 4069 |


---

|  |  |
| --- | --- |
| Agilent ID | A\_43\_P10020 |
|  | A\_24\_P42264 |
|  | A\_14\_P103181 |
|  | A\_23\_P76192 |
|  | A\_42\_P773657 |


---

|  |  |
| --- | --- |
| Cellular Localization | Mitochondrion |
|  | Extracellular region |
|  | Cytoplasm |
|  | Organelle |
|  | Cell |


---

|  |  |
| --- | --- |
| Pathway | Zn xs inventory |
|  | Zn xs DIN |


---

|  |  |
| --- | --- |
| GO Process | carbohydrate metabolism |
|  | cell wall catabolism |
|  | cytolysis |
|  | defense response to bacteria |


---

|  |  |
| --- | --- |
| UniGene | Rn.2283 |
|  | Hs.524579 |


---

|  |  |
| --- | --- |
| Affymetrix Probeset ID | 1370154\_at |
|  | 1555745\_a\_at |
|  | Hs2.234734.3.S1\_3p\_s\_at |
|  | J03801\_f\_at |
|  | M19045\_f\_at |
|  | M21119\_s\_at |
|  | rc\_AA892775\_at |
|  | X14008\_rna1\_f\_at |
|  | 213975\_s\_at |
|  | 35926\_s\_at |
|  | Hs.277431.0.S1\_3p\_s\_at |
|  | RC\_AA347359\_s\_at |


---

|  |  |
| --- | --- |
| EC Number | EC 3.2.1.17 |


---

|  |  |
| --- | --- |
| GO Function | hydrolase activity |
|  | hydrolase activity, acting on glycosyl bonds |
|  | lysozyme activity |
|  | catalytic activity |


---

|  |  |
| --- | --- |
| Nucleotide | AK130127 |
|  | AK130149 |
|  | BC058490 |
|  | J03801 |
|  | M21119 |
|  | L12458 |
|  | NM\_000239 |
|  | BC004147 |
|  | L12459 |
|  | U25677 |
|  | X14008 |
|  | M19045 |
|  | NM\_012771 |


---

|  |  |
| --- | --- |
| Protein | AAA36188 |
|  | NP\_000230 |
|  | AAH04147 |
|  | CAA32175 |
|  | AAA41552 |
|  | NP\_036903 |
|  | AAH58490 |
|  | P00697 |
|  | AAA41551 |
|  | AAA59535 |
|  | Q05820 |
|  | AAC63078 |
|  | P61626 |
|  | AAA59536 |


---

|  |  |
| --- | --- |
| Organism | Mammal |


---

|  |  |
| --- | --- |
| Location | chromosome 7, 7q22 (Rattus norvegicus) |
|  | chromosome 12, 12q15 (Homo sapiens) |


---

|  |  |
| --- | --- |
